# Relationship between epicardial adipose tissue volume and atrial fibrillation in patients with rheumatoid arthritis

**DOI:** 10.3389/fcvm.2025.1508025

**Published:** 2025-04-09

**Authors:** Hao Yao, Jian Chen, Xueling Li, Xin Zhang

**Affiliations:** Heart Center, Department of Cardiovascular Medicine, Zhejiang Provincial People’s Hospital (Affiliated People’s Hospital), Hangzhou Medical College, Hangzhou, Zhejiang, China

**Keywords:** epicardial adipose tissue, rheumatoid arthritis, atrial fibrillation, left atrial diameter, CHA_2_DS_2_-VASc scores

## Abstract

**Introduction:**

Epicardial adipose tissue (EAT) is involved in cardiac inflammatory responses and has been associated with both atrial fibrillation (AF) and rheumatoid arthritis (RA). However, the condition of EAT in patients with both RA and AF is still unclear. In addition, the risks of stroke and bleeding in patients with both RA and AF are unknown.

**Methods:**

A retrospective analysis was conducted in patients with RA aged ≥18 years from August 2021 to May 2023, and compared with age- and gender-matched patients without RA. The volume of EAT was measured using chest computed tomography and the EAT/body mass index (BMI) ratio was used to correct for the possible impact of BMI differences. The stroke and bleeding risks of the patients were assessed using the CHA_2_DS_2_-VASc or HAS-BLED scores.

**Results:**

A total of 145 patients with RA and 282 patients without RA were included. The volume of EAT or EAT/BMI ratio was similar between the patients with RA and no AF and those without both RA and AF. Compared to the patients without AF, those with AF had a larger EAT volume or EAT/BMI ratio, regardless of whether they had RA or not. EAT/BMI ratio was significantly associated with left atrial (LA) diameter among the patients with RA (RR = 2.23, *P* < 0.001) but not among the patients without RA (*P* < 0.954). The RA groups had larger LA-EAT volume (31.53 ± 11.02 mm^3^ vs. 22.56 ± 9.58 mm^3^, *p* < 0.001) and LA-EAT/Total EAT ratio (23.02% ± 3.62% vs. 18.74 ± 3.38 mm^3^, *p* < 0.001) than that in non-RA groups. In addition, the proportion of patients with high stroke risk scores was higher among the patients with both RA and AF compared to those without RA but with AF (90.90% vs. 72.00% in men; 84.78% vs. 71.11% in women), while the proportion of patients with high bleeding risk scores was lower (22.06% vs. 27.85%).

**Conclusion:**

LA diameter correlates with the EAT/BMI ratio in patients with RA who exhibit larger LA-EAT volume and LA-EAT/total EAT ratios compared to individuals without RA.

## Introduction

Atrial fibrillation (AF) is one of the most common arrhythmias in the world, but the pathogenesis of AF is not fully understood. Recent research has found a close link between inflammation and atrial fibrillation (1). Systemic inflammation, such as rheumatoid arthritis (RA), ankylosing spondylitis, Crohn's disease, and psoriasis, may promote atrial fibrillation and other cardiac arrhythmias (2). Compared with patients without rheumatoid arthritis, those with rheumatoid arthritis have a 30% increased risk of developing atrial fibrillation (3). There is a strong association between inflammatory markers, particularly tumor necrosis factor alpha (TNFα), interleukin 6 (IL-6), and C-reactive protein (CRP), and the risk of AF (4).

Epicardial adipose tissue (EAT) is a fat layer located between the myocardium and the visceral pericardium. EAT serves as a source of inflammatory mediators. In patients undergoing coronary artery bypass grafting (CABG), there is a significant increase in the mRNA and protein levels of IL-1β, IL-6, monocyte chemoattractant protein 1 (MCP-1), and TNFα within the EAT reservoir (5). Moreover, EAT is associated with the occurrence, severity, and recurrence of AF. For every one standard deviation increase in EAT volume, the risk of developing AF is up to 2.6 times higher, with the risks for developing paroxysmal and persistent AF being 2.1 times and 5.4 times higher, respectively (6, 7). Patients with RA have thicker EAT compared to the general population (8). In patients with RA, epicardial adipose tissue volume (EATv) is correlated with a greater burden of coronary artery plaque and the presence of vulnerable plaque characteristics (9). However, there are currently no studies on EAT in patients with RA who also have atrial fibrillation.

Compared to sinus rhythm, atrial fibrillation increases the risk of stroke by four to five times (10). Such a situation is more pronounced in patients with rheumatoid arthritis, especially in younger patients (11). Anticoagulation therapy can reduce the risk of ischemic stroke and systemic embolic events in patients with atrial fibrillation. Studies have found that the proportion of patients with rheumatoid arthritis who also have atrial fibrillation and receive anticoagulation therapy is not optimal (12). However, there have been no studies to explore the bleeding risks associated with anticoagulation therapy in patients with rheumatoid arthritis and atrial fibrillation.

The purpose of this study was to compare the differences in EAT volume between patients with rheumatoid arthritis who also have atrial fibrillation and those with atrial fibrillation without rheumatoid arthritis and to assess the differences in stroke risk and bleeding risk between the two groups.

## Methods

### Study population

This retrospective observational study included patients who presented between August 2021 and May 2023. All the enrolled participants had been diagnosed with RA according to the 2010 American College of Rheumatology/European League Against Rheumatism (ACR/EULAR) criteria and were ≥18 years old. Furthermore, we screened age- and gender-matched patients without RA as controls. Patients for whom chest computed tomography (CT) data could not be obtained were excluded. Clinical characteristics including age, gender, body mass index (BMI, kg/m^2^), known diagnosis of hypertension, diabetes mellitus (DM), heart failure, and echocardiography were recorded. A patient was considered to have hypertension if they reported either a diagnosis of hypertension and/or the use of an antihypertensive drug or had been newly diagnosed with systolic blood pressure ≥140 mmHg and/or diastolic blood pressure ≥90 mmHg in three measurements made on different days. DM was defined as being pre-diagnosed and/or being on antidiabetic treatment. Heart failure included heart failure with reduced ejection fraction (HFrEF), heart failure with mildly reduced ejection fraction (HFmrEF), and heart failure with preserved ejection fraction (HFpEF). The study was approved by the Clinical Medical Ethics Committee of Zhejiang Provincial People's Hospital and was performed in line with the principles of the Declaration of Helsinki.

### EAT measurement

EAT was quantified on routine chest CT images. The volume of EAT was measured by 3D Slicer 5.6 software. Fat voxels were defined using a threshold attenuation value of −150 to −50 Hounsfield Units (HU). EAT was separated from pericardial fat by manually tracing a single region of interest along the pericardium on each slice. The voxels in each slice were summed to determine the total and periatrial EAT volumes. The measured area for total EAT was from the inferior surface of the origin of the left pulmonary artery to the apex of the left ventricle. Thereafter, the pericardial adipose tissue in the left ventricle in front of the mitral annulus, the epicardial adipose tissue in the right atrium in front of the right superior pulmonary vein, and the epicardial adipose tissue below the plane of the coronary sinus were manually removed, and the remaining epicardial adipose tissue was defined as left atrial EAT (LA-EAT). Segmentation and extraction of radiomic parameters were performed by an investigator blinded to the clinical information of the patients. Only adipose tissue within the pericardial sac was studied. The volume of EAT in the selected regions was directly calculated by the software.

### Risk scores

Stroke risk factors in the patients with AF were evaluated using CHA_2_DS_2_-VASc scores (13). Oral anticoagulant (OAC) treatment is recommended with a CHA_2_DS_2-_VASc score ≥2 (men) or ≥3 (women). The HAS-BLED score (13) was used to assess the bleeding risk of the enrolled patients with AF. Participants with a HAS-BLED score ≥3 were considered to have a higher bleeding risk when receiving OAC treatment.

### Statistical analysis

Continuous variables are given as mean ± SD and compared using *t*-tests. Categorical variables are given as frequency (proportion) and compared using the chi-square test or Fisher's exact test. A two-tailed *p*-value of <0.05 was considered statistically significant. The association between the EAT/BMI ratio and LA diameter was evaluated with multiple non-linear regression. Statistical analyses were performed using SPSS version 26.0.

## Results

### Study population

In total, 145 patients with RA and 282 age- and gender-matched patients without RA were enrolled for further analysis. The clinical characteristics of the enrolled patients are summarized in Table 1. Compared to the patients without RA, the BMI of the patients with RA was slightly lower (22.80 ± 3.56 kg/m^2^ vs. 23.70 ± 3.37 kg/m^2^, *p* = 0.020). Traditional cardiac risk factors, such as hypertension, diabetes mellitus, and heart failure, were similarly distributed among the patients with RA and those without RA. Echocardiographic characteristics, including LA diameter and left ventricular ejection fraction (LVEF), were not statistically different between the groups. Moreover, there was no statistically significant difference in the overall HAS-BLED scores between the two groups (1.97 ± 1.18 vs. 1.94 ± 1.25, *p* = 0.907), while the CHA_2_DS_2_-VASc scores were higher in the patients with RA patients than in those without RA (4.03 ± 1.87 vs. 3.47 ± 1.81, *p* = 0.047).

**Table 1:** Clinical characteristics of patients with and without RA.

Variable	RA (*n* = 145)	Non-RA (*n* = 282)	*p*-value
Age (years)	70.08 ± 11.44	69.58 ± 11.58	0.801
Female	99 (68.28%)	183 (64.89%)	0.485
Hypertension	77 (53.10%)	139 (49.29%)	0.456
Diabetes mellitus	17 (11.72%)	41 (14.54%)	0.421
Body mass index (kg/m^2^)	22.80 ± 3.56	23.70 ± 3.37	0.020
Heart failure	51 (35.17%)	81 (28.72%)	0.172
LA diameter (mm)	43.89 ± 7.11	41.53 ± 7.23	0.111
LVEF (%)	54.52 ± 4.12	55.38 ± 4.86	0.069
CHA_2_DS_2_-VASc score	4.03 ± 1.87	3.47 ± 1.81	0.047
HAS-BLED score	1.97 ± 1.18	1.94 ± 1.25	0.907

LA diameter, left atrial diameter; LVEF, left ventricular ejection fraction.

### Association between EAT volume and AF in patients with RA and patients without RA

**Figure 1:**
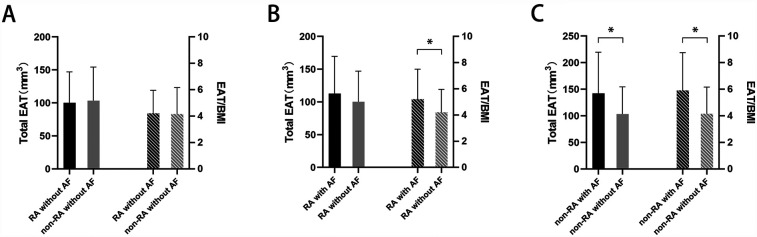
Total EAT volume or EAT/BMI ratio in **(A)** patients with RA but without AF vs. those without both RA and AF, **(B)** patients with both RA and AF vs. those with RA but without AF, and **(C)** patients without RA but with AF vs. those without both RA and AF. *p < 0.05. EAT, epicardial adipose tissue; BMI, body mass index; RA, rheumatoid arthritis; AF, atrial fibrillation.

Due to the difference in BMI between the patients with RA and those without RA, and the potential influence of BMI on EAT volume (14), we assessed both the total EAT volume and the EAT volume normalized by BMI. There were no statistically significant differences in either total EAT or the EAT/BMI ratio between the patients with RA and no AF and those without RA and no AF (100.20 ± 46.93 mm^3^ vs. 103.40 ± 51.08 mm^3^, *p* = 0.729; 4.21 ± 1.74 vs. 4.16 ± 2.00 mm^3^, *p* = 0.875) (Figure 1A). Compared to the patients with RA and no AF, the patients with both RA and AF did not show statistical differences in total EAT (112.80 ± 56.74 mm^3^ vs. 100.2 ± 46.93 mm^3^, *p* = 0.159) (Figure 1B), but had a higher EAT/BMI ratio (5.20 ± 2.30 vs. 4.21 ± 1.74, *p* = 0.007). However, among the patients without RA, patients without RA but with AF had higher levels of both total EAT and EAT/BMI compared to those without both RA and AF (142.20 ± 77.43 mm^3^ vs. 103.40 ± 51.08 mm^3^, *p* < 0.001; 5.91 ± 2.84 vs. 4.16 ± 2.00, *p* < 0.001) (Figure 1C).

### Association between LA size and LA-EAT volume and total EAT in patients with RA and patients without RA

**Figure 2:**
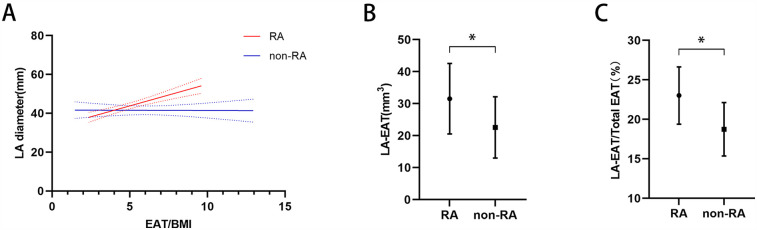
**(A)** Association between LA diameter and the EAT/BMI ratio in patients with RA and patients without RA. LA-EAT volume **(B)** and the LA-EAT/total EAT ratio **(C)** among patients with RA and patients without RA. *p < 0.05. LA diameter, left atrial diameter; EAT, epicardial adipose tissue; BMI, body mass index; RA, rheumatoid arthritis.

Non-linear regression analysis showed that EAT/BMI was significantly associated with LA diameter among the patients with RA (RR = 2.23, 95%CI: 1.46–3.00, *P* < 0.001) but not among those without RA (RR = −0.02, 95%CI: −0.81 to 0.77, *P* < 0.954) (Figure 2A). Among the patients with AF, the RA group had larger LA-EAT volume (31.53 ± 11.02 mm^3^ vs. 22.56 ± 9.58 mm^3^, *p* < 0.001) and LA-EAT/total EAT ratio (23.02% ± 3.62% vs. 18.74 ± 3.38 mm^3^, *p* < 0.001) than those in the non-RA group (Figures 2B,C).

### Stroke and bleeding scores in AF patients

According to the CHA_2_DS_2_-VASc score, 20 (90.90%) male and 39 (84.78%) female patients with RA had a high risk of stroke (score ≥2 in men and ≥3 in women). Among the patients without RA, the number of patients with a high stroke risk was 36 (72.00%) for the men and 64 (71.11%) for the women (Table 2). A HAS-BLED score of ≥3 points indicates a high risk of bleeding. Among the 208 patients with AF, 41 were classified as being at high risk for bleeding. Specifically, there were 15 (22.06%) with a high bleeding risk among the patients with both RA and AF, while among those without RA but with AF, there were 39 (27.85%) (Table 2).

**Table 2:** CHA_2_DS_2_-VASc and HAS-BLED scores in patients with AF with or without RA.

	RA with AF	Non-RA with AF
CHA_2_DS_2_-VASc score		
≥2 in men	20 (90.90%)	36 (72.00%)
≥3 in women	39 (84.78%)	64 (71.11%)
HAS-BLED score		
≥2	15 (22.06%)	39 (27.85%)

## Discussion

In this study, we investigated the relationship between EAT volume, RA, and AF. Patients with AF, regardless of whether they had RA or not, had a higher EAT volume than those without AF. The EAT/BMI ratio was associated with LA diameter among the patients with RA. Compared to the patients without RA but with AF, those with both RA and AF had a larger LA-EAT volume. Furthermore, we also studied the risk of stroke and bleeding in patients with AF. Compared to those without RA, the proportion of patients with both RA and AF who had a high CHA_2_DS_2_-VASc score (≥2 for males or ≥3 for females) was higher, while the proportion of those with a HAS-BLED score of ≥3 was not higher than that of the patients without RA but with AF. This indicates that patients with both RA and AF may have a greater need for anticoagulation and a lower bleeding risk.

### The role of EAT in both rheumatoid arthritis and atrial fibrillation

The role of EAT in systemic inflammatory responses is gradually gaining attention. EAT is in direct contact with the superficial myocardium and shares a microcirculatory system, thus it can influence cardiac inflammation through various mechanisms. EAT is one of the sources of cardiac inflammatory mediators. In patients undergoing CABG, the expression of IL-1β, IL-6, MCP-1, and TNF*α* mRNA and protein in the EAT is significantly elevated (5). Previous research has found that the thickness of EAT in patients with RA is greater than in the control population (8). In our study, there was no difference in EAT volume between patients with RA and those without RA, which is consistent with the results of Karpouzas et al. (9). This may be due to the impact of the measurement method, as echocardiography usually only measures the thickness of EAT on the free wall of the right ventricle, while CT or MRI can measure the volume of EAT over the entire cardiac surface (15).

Although the pathogenesis of AF remains a subject of debate, the inflammatory mechanism has gained widespread recognition in recent years. RA increases the risk of AF by 30% compared to patients without RA (3). After adjusting for cardiovascular confounding factors, the risk of AF is still elevated (16, 17). EAT is involved in cardiac inflammatory responses and is closely related to AF (18). In our study, after adjusting for the potential impact of BMI, we found that the patients with AF, whether with or without RA, had a larger EAT volume, further indicating that AF itself has an independent inflammatory pathogenic process.

Atrial structural remodeling is associated with the maintenance and recurrence of atrial fibrillation. Our research found that in patients with both RA and AF, the LA anteroposterior diameter is associated with the EAT/BMI ratio. As the EAT/BMI ratio increases, the LA diameter enlarges, a phenomenon not observed in patients with atrial fibrillation but without RA. Previous case reports have documented extensive atrial fibrosis in patients with RA (19). Diffuse atrial fibrosis reduces left atrial compliance, leading to left atrial dilation. Furthermore, compared to patients without RA, those with RA have more pericardial adipose tissue surrounding the left atrium. Chahine et al. (20) found that left atrial EAT is independently associated with left atrial volume and fibrosis, and that the location of EAT does not colocalize with the region of atrial fibrosis, suggesting that EAT may promote atrial structural remodeling through the paracrine pathway of inflammatory factors.

### The stroke and bleeding scores in patients with both RA and AF

Compared to those without RA but with AF, a larger proportion of the patients with both RA and AF were at high risk of stroke (CHA_2_DS_2_-VASc score ≥2 in men or ≥3 in women). This means that more patients with both RA and AF require anticoagulation therapy. Moreover, the recurrence rate of AF after catheter ablation is higher in patients with RA than in those without RA (21). Therefore, a more aggressive anticoagulation strategy should be adopted for patients with both RA and AF. However, according to a survey by Semb et al. in 2022 (12), anticoagulation treatment among patients with both RA and AF globally was not at an optimal level, with only 64.3% of patients who met the indications for anticoagulation receiving such treatment. Concerns about the bleeding risks associated with anticoagulation therapy may be a contributing factor.

Unexpectedly, our study found that patients with both RA and AF had a lower risk of bleeding than those without RA, which seems to contradict the conventional view. We speculate that this may be because our study used the HAS-BLED score to assess the risk of bleeding. The “drug” component of the HAS-BLED score only evaluates antiplatelet drugs or non-steroidal anti-inflammatory drugs (NSAIDs) (22) but does not include corticosteroids and other anti-rheumatic drugs that may lead to bleeding complications. Therefore, the results of this retrospective study may not directly reflect the true bleeding situation among patients with both RA and AF.

### Limitations

This study had several limitations. First, our study sample size was relatively small, especially with fewer male patients with RA, which may bias the results. Second, due to the design of the retrospective study, the assessment of stroke and bleeding risks does not directly represent the actual situation in the real world. Future prospective studies are needed to further verify the results.

## Conclusion

LA diameter was associated with the EAT/BMI ratio among patients with RA. Compared to patients without RA, those with RA had a larger LA-EAT volume and LA-EAT/total EAT ratio.

## Data Availability

The original contributions presented in the study are included in the article/Supplementary Material, further inquiries can be directed to the corresponding author.
